# Determination of Lead Employing Simple Flow Injection AAS with Monolithic Alginate-Polyurethane Composite Packed In-Valve Column

**DOI:** 10.3390/molecules26154397

**Published:** 2021-07-21

**Authors:** Piyanat Issarangkura Na Ayutthaya, Chonnipa Yeerum, Kullapon Kesonkan, Kanokwan Kiwfo, Kate Grudpan, Norio Teshima, Hiroya Murakami, Monnapat Vongboot

**Affiliations:** 1 Department of Chemistry, Faculty of Sciences, King Mongkut’s University of Technology Thonburi, Bangkok 10140, Thailand; piyanat.tp@gmail.com (P.I.N.A.); chonnipa.yeerum@gmail.com (C.Y.); kullapon.kesonkan@gmail.com (K.K.); 2 Center of Excellence for Innovation in Analytical Science and Technology and Department of Chemistry, Faculty of Sciences, Chiang Mai University, Chiang Mai 50200, Thailand; k.kanokwan11@gmail.com; 3 Department of Applied Chemistry, Aichi Institute of Technology, 1247 Yachigusa, Yakusa-cho, Toyota 470-0392, Japan; teshima@aitech.ac.jp (N.T.); hmurakami@aitech.ac.jp (H.M.)

**Keywords:** lead(II), monolithic column, alginate-polyurethane composite, flow injection, FlameAAS, in-valve column

## Abstract

A simple flow injection FlameAAS for lead determination with an alginate-polyurethane composite (ALG-PUC) monolithic in-valve column has been developed. The ALG-PUC monolithic rod was prepared by mixing methylene diphenyl diisocyanate with polyol and sodium alginate with the ratio of 2:1:1 by weight for a 5 min polymerization reaction. It was then put into a column (0.8 cm i.d × 11 cm length) situated in a switching valve for the FI set up. A single standard calibration could be obtained by plotting the loaded µg Pb^2+^ vs. FI response (absorbances). The loaded µg Pb^2+^ is calculated: μg Pb^2+^ = FR_load_ × LT × C_Pb_^2+^, where the FR load is the flow rate of the loading analyte solution (mL min^−1^), LT is the loading time (min), and C_Pb_^2+^ is the Pb^2+^ concentration (µg mL^−1^). A linear calibration equation was obtained: FI response (absorbances) = 0.0018 [µg Pb^2+^] + 0.0032, R^2^ = 0.9927 for 1–150 µg Pb^2+^, and RSD of less than 20% was also obtained. Application of the developed procedure has been demonstrated in real samples.

## 1. Introduction

Lead is a highly toxic element that can be found in the environment. For chronic exposure, it can harm and affect humas health, for example, it can cause nephritis of the kidney, brain damage, and central nervous system disorders [1]. Atomic absorption spectrometry (AAS) is among one of the various methods for the determination of lead due to its availability in laboratories and high selectivity in the detection of Pb^2+^ [2,3,4]. Other techniques employed as standard methods include, electrothermal atomic absorption, inductively coupled plasma, inductively coupled plasma/mass spectrometry, anodic stripping voltammetry, and colorimetric employing dithizone [5]. Adsorptive stripping voltammetry (AdSV) provides good sensitivity (ng mL^−1^ or lower [6,7,8]) and could be used for multi-elements analysis [9,10,11]. ICP-MS or ICP-AES also provides similar information [12,13,14,15]. The cost of operation in addition to the cost of the instrument itself would be relatively high, especially in less developed places. A well-trained person is needed for its operation. A flame atomic absorption spectrometer (FlameAAS) is commonly available in most laboratories. There have been a number of works applying the flow techniques to FlameAAS to enhance performance [16,17,18,19,20,21]. In our research group, we have employed it with polyurethane foam (PUF) for sample pretreatment because of its various advantages [22,23]. The use of PUF with flow injection analysis (FIA) has been reported as modified-PUF powder for ions adsorption in online systems [20,21,24,25,26], but some disadvantages such as high pressure when the system was used over a long period of time and inconvenience when packing the material into the column were encountered. For the modification of PUF with alginate, it has shown selectivity toward Pb^2+^, durability, and reusability [27]. Advantages obtained using a monolithic PUF rod have been observed in previous work on SDS assay [28]. Instead of using the previously reported alginate-polyurethane composite (ALG-PUC) in powder form as a batch procedure for removal of lead, the grafting of alginate and PUF was synthesized as a modified ALG-PUC monolithic column situated in-valve during FI set up. It is expected that a long system running time with low back pressure was beneficial due to the high porosity of ALG-PUC, and it was easy to fabricate. From our previous experiences, using an in-valve column for FI setup made the determination of Pb^2+^ while employing single standard calibration possible, even without using a monolith column [29]. This work aimed to prove that the use of a monolithic ALG-PUC in-valve column with single standard calibration approach can enhance the performance of FlameAAS in lead determination, although with limitations the in availability of the instrument components.

## 2. Results and Discussion

### 2.1. Synthesis of Polyurethane Foam (PUF) and Alginate-Polyurethane Composite (ALG-PUC) and Their Characteristics

**Figure 1:**
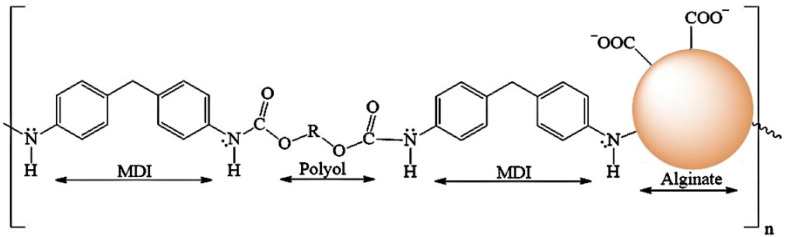
The estimated ALG-PUC structure.

The chemical structure of PUF consists of diisocyanate groups from MDI and hydroxyl groups from polyol [-MDI-Polyol-MDI-Polyol-]_n_, while the components of ALG-PUC are the diisocyanate groups from the MDI and hydroxyl groups from polyol and alginate [-MDI-Polyol-MDI-ALG-]_n_ (Figure 1). The characterization of PUF and ALG-PUC was investigated using IR spectra (see Figure S1) and SEM images (see Figure S2). Alginate might have a role as a hydroxyl group and may bond with diisocyanate groups. In contrast ALG-PUC is whiter and has a higher porosity than PUF (Figure S3). Alginate appears on the binding side of the carboxylic group (-COO^−^) for the sorption of Pb^2+^ as an ion-association, which is highly selective with Pb^2+^ ions when the pH is higher than 3.96 [27]. The ratio of the carboxylic group and Pb^2+^ ions was 2:1 [27]. Monolithic ALG-PUC could only be used for at least 75 cycles, as the absorbance would decrease significantly after that.

### 2.2. Study of Elution Profile of Monolithic ALG-PUC Packed In-Valve-FI

**Figure 2:**
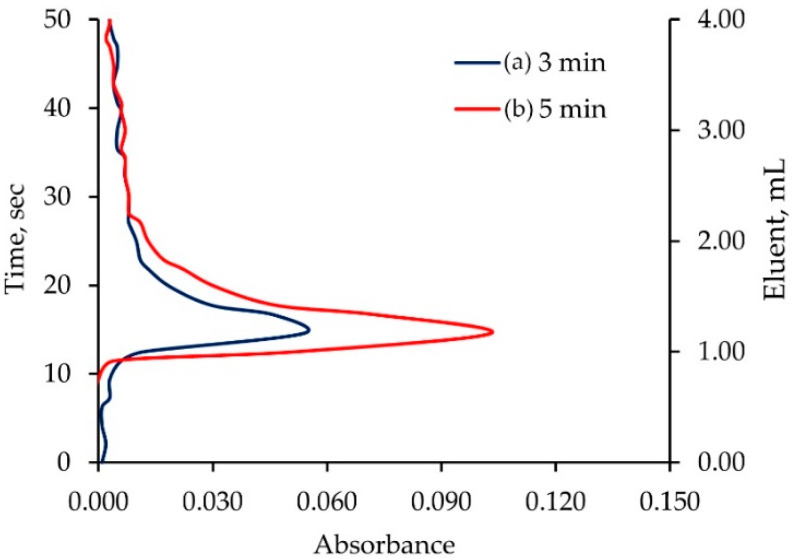
Elution profiles of loaded Pb^2+^ on the monolithic ALG-PUC in-valve column situated in FI set up: (a) 3 min and (b) 5 min loading time.

The elution of a monolithic ALG-PUC column for Pb^2+^ was studied. An in-valve column (0.8 cm i.d × 11 cm length) packed with monolithic ALG-PUC was situated in a switching valve of the FI set up (see in the Section 3.1). A 10 µg mL^−1^ Pb^2+^ solution was passed through the column for 3 min. The residual and unsorbed Pb^2+^ was cleaned with water. The column was eluted with 2 mol L^−1^ nitric acid. The experiments for a 5 min loading time were also performed. The obtained elution profiles are illustrated in Figure 2. It can be seen that an eluent (2 mol L^−1^ nitric acid) volume of 4 mL (flow rate 5 mL min^−1^, with less than 1 min) could practically quantitatively elute the loaded Pb^2+^. The peak maxima were observed in the same position. This indicated that the peak height could be used for the FI-response corresponding to absorbance due to the sorbed amount of lead.

### 2.3. Effect of Nitric Acid Concentration

**Figure 3:**
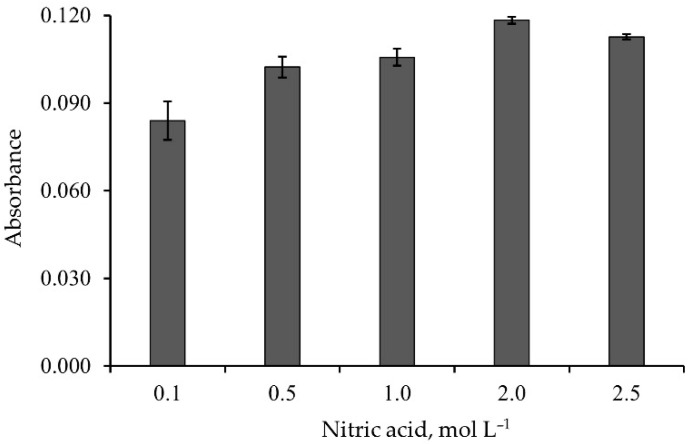
Effect of nitric acid concentration on elution of the loaded Pb^2+^.

A 60 µg of Pb^2+^ was loaded into the monolithic ALG-PUC column. Elution using different concentrations (0.1–2.5 mol L^−1^) of nitric acid was studied. The results are illustrated in Figure 3. A concentration of 2 mol L^−1^ or above yielded the maximum elution of Pb^2+^. This could be because the H^+^ from nitric acid would replace Pb^2+^ at the binding site in the connection with carboxyl group (-COO^−^) of alginate [27].

### 2.4. Single Standard Calibration

**Figure 4:**
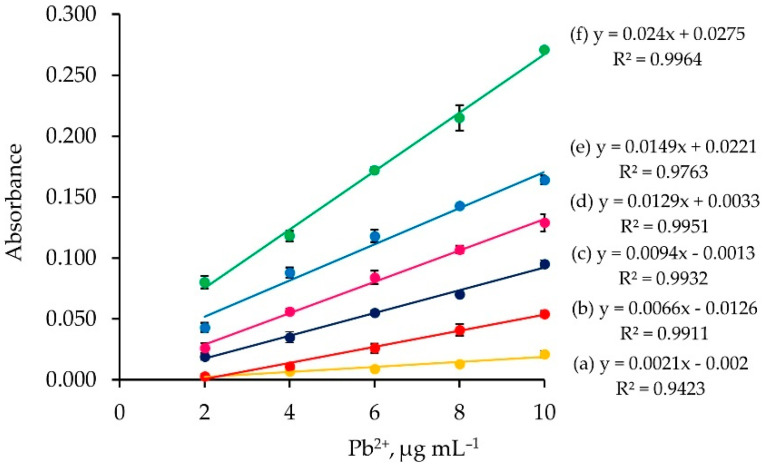
Calibration graphs obtained for different loading times: (a) 2 min; (b) 3 min; (c) 5 min; (d) 7 min; (e) 10 min; and (f) 15 min.

A series of Pb^2+^ solutions (2.0–10.0 µg mL^−1^) were passed into the column with various loading times (2–15 min) and under the same loading flow rate (1 mL min^−1^) and elution condition (2 mol L^−1^ nitric acid, 5 mL min^−1^). Linear calibration graphs based on each loading time were obtained (plot of absorbance vs. Pb^2+^ concentration), as displayed in Figure 4. It can be seen that the longer the loading time, the higher the calibration slope.

One can calculate the microgram of loaded Pb^2+^ by µg Pb^2+^ = FR_load_ × LT × C_Pb_^2+^, where FR_load_ is the flow rate of loading analyte solution (mL min^−1^), LT is the loading time (min), and C_Pb_^2+^ is the Pb^2+^ concentration (µg mL^−1^). It can be observed from Figure 4 that the same amount of loaded Pb^2+^ exhibits the same absorbance values.

**Figure 5:**
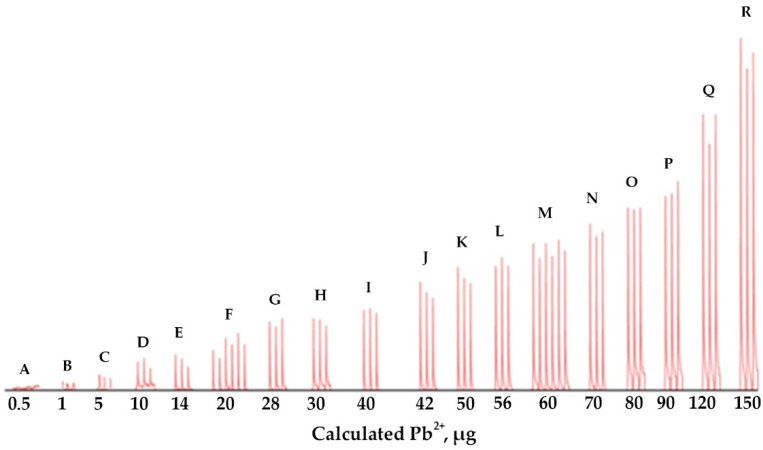
FI responses due to various amounts of Pb^2+^. (See Table 1).

**Figure 6:**
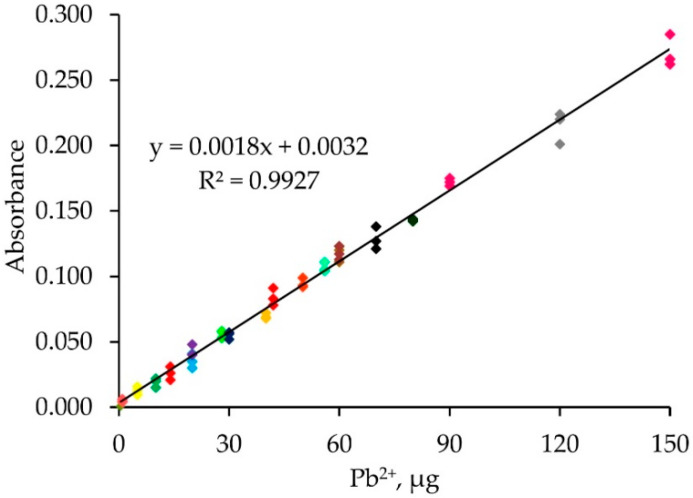
The single standard calibration graph.

A single calibration approach was investigated. Solutions of different Pb^2+^ concentrations (0.1–10.0 µg mL^−1^) were percolated into the monolithic ALG-PUC packed in-valve of the FI set up for different loading times (2–15 min) with a loading flow rate of 1 mL min^−1^ and with the same elution condition as before. The results are presented in Table 1 and Figure 5. As expected, the same microgram of Pb^2+^ (from the calculation described previously) provided practically the same FI response (absorbances), for example, for the condition of F, 20 µg Pb^2+^ obtained from either using Pb^2+^ 2 or 4 µg mL^−1^ with a loading time of 5 or 10 min, respectively, provided FI responses (absorbances) of 0.035 ± 0.004 and 0.043 ± 0.004. Similarly, for the conditions M, 60 µg Pb^2+^ resulted from using either 4 or 6 µg mL^−1^ Pb^2+^ with a 10 or 15 min loading time produced 0.118 ± 0.005 and 0.118 ± 0.004 FI responses, respectively. A single standard calibration can be then obtained by plotting the calculated loaded µg Pb^2+^ vs. FI response (absorbances), as illustrated in Figure 6, with linear calibration equation being FI response (absorbances) = 0.0018 [µg Pb^2+^] + 0.0032, R^2^ = 0.993 for 1–150 µg Pb^2+^. The relative standard deviation was less than 20%. If a loading higher than 150 µg Pb^2+^ was applied, the saturation of the ALG-PUC binding site was situated. With limitations of the instrumentation that was available to our lab group explained above, we needed to set up a system using only one single injection valve and a peristatic pump. With that setup, which was described earlier in the manuscript, the same calculated µg Pb^2+^ (from flow rate, loading time, and concentration) was loaded onto the column and provided the same FI response, leading to the validity of the single standard calibration. With the setup (only very basic components were available for our work), to result in the applicability of the single standard calibration approach, the loading time needed to be 5 min or longer (if a time shorter than 5 min, there would be an error due to switching the valve for flow lines, see Table S1), while the flow rate was fixed at 1 mL min^−1^. Considering for loading time of 5 min for 1 µg Pb^2+^ (calculated), which was the last point of the calibration, a 1 mL min^−1^ flow rate, would result in 0.2 µg mL^−1^ Pb^2+^ of loading solution. This would reflect the limit of quantitation. The sensitivity obtained by the setup could be improved if the system could be composed of better-quality components: a peristatic pump and a three way valve with automation control. The concept should further be developed for automation using a sequential injection system. It should be noted that using the single standard approach for a given set of conditions (fixed flow rate), if loading a sample solution with a given loading time produced a FI response lower than the lowest point of the linear calibration, the sample solution could be reloaded with more appropriate time to produce an FI response within the linear range. Similarly, if loading a sample solution resulted in a higher FI response, a shorter reloading time would provide a FI response within the linear range.

### 2.5. Lead Assay in Samples of Aqueous Sulfuric Acid Solutions Contained in Acid Batteries

The developed method was applied to determine the amount of Pb^2+^ in samples of aqueous sulfuric acid solutions contained in acid batteries. The results of the recovery studies are represented in Table 2.

## 3. Materials and Methods

### 3.1. Apparatus

In this work, an atomic absorption spectrometer (PerkinElmer, AAnalyst 800, Waltham, MA, USA) was previously used. Unfortunately, due to the accidental failure of this FlameAAS, the change to the new one was inevitable. For this reason, an atomic absorption spectrometer (Hitachi, ZA 3300, Tokyo, Japan) was selected and tested for the experimental conditions before use. The instrument provided similar results. A Fourier-transform infrared spectrometer (Thermo Scientific, Nicolet 6700, Waltham, MA, USA) and scanning electron microscope, SEM, (JEOL, JSM-6610 LV, Tokyo, Japan) were to characterize the synthesized monolithic ALG-PUC column. Six-port valves (Rheodyne, 7125, Berkeley, CL, USA), a peristaltic pump (Cole-Parmer, Masterflex L/S, Vernon Hills, IL, USA) for Pump 1 and a peristaltic pump (Cole-Parmer, Masterflex L/X, Vernon Hills, IL, USA) for Pump 2 were included. A digital pH meter (METTLER TOLEDO, Greifensee, Switzerland) was for pH adjustment.

**Figure 7:**
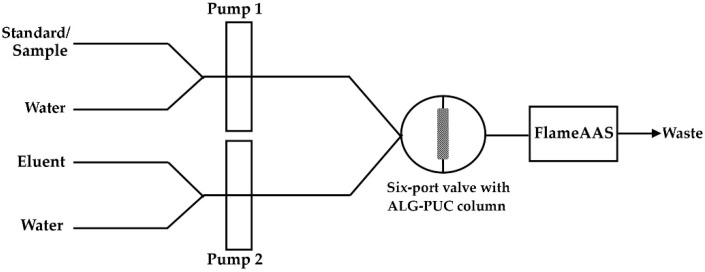
FI-FlameAAS system.

Due to the use of a single six-port valve, the FI setup with an in-valve column for the determination of lead using FlameAAS was designed as shown in Figure 7. Water used as a carrier was passed through Pump 2 into the column at a flow of 5 mL min^−1^, it then passed through the FlameAAS, while a standard/sample solution was passed through Pump 1 into the six-port valve, finishing as waste (period d in Figure S4). Next, the Pb^2+^ standard or sample solution was delivered with a 1 mL min^−1^ flow rate to the in-valve monolithic ALG-PUC column via Pump 1 with a certain loading time (period a in Figure S4). Pump 2 would deliver water to wash the residual and unsorbed Pb^2+^ with a flow rate of 5 mL min^−1^ for 1 min (period b in Figure S4). At that stage, the valve was in the injection position. Water from Pump 1 passed the six-port valve to waste. The valve was then changed into the loading position (period x1 in Figure S4). The eluent from Pump 2 passed into the six-port valve. After that, the valve was changed to its injection position (period c in Figure S4), the sorbed Pb^2+^ was eluted from the column using 2 mol L^−1^ nitric acid via Pump 2 with a 5 mL min^−1^ flow rate for 1 min and absorbance was detected by the FlameAAS. The valve was changed to loading position. The eluent line was switched to water (period x2 in Figure S4). The washing step was applied (period d was repeated).

### 3.2. Reagents and Materials

All of the reagents used in this work were of analytical grade. Deionized water made of the volume of the solutions. Working solutions of Pb^2+^ were prepared from 1000 µg mL^−1^ Pb^2+^ stock solution (Loba chemie, Mumbai, India).

### 3.3. Preparation of Monolithic ALG-PUC Rod

The mixing of methylene diphenyl diisocyanate (MDI; IRPC, Rayong, Thailand) with polyol (polyether; IRPC, Rayong, Thailand) and sodium alginate in the ratio of 2:1:1 by weight and polymerization was completed within 5 min to obtain a bulk product in a 500 mL beaker. A monolithic ALG-PUC rod was obtained by pressing a plastic rod (0.8 cm i.d × 11 cm length) into the bulk product. The obtained monolithic rod was about 0.1 g in weight (see Figure S3) and was put into an acrylic rod (0.8 cm i.d × 11 cm length) that had ferules at the two ends with tubing connecting it into the flow system.

### 3.4. Sample Preparation

Each of the aqueous sulfuric acid solutions contained in acid batteries was filtrated and pipetted 10.00 mL. After that, the adjustment to pH 4 by 10 M NaOH was applied. Finally, deionized water was added to the treated sample to add volume up to 50.00 mL.

## 4. Conclusions

The grafting of alginate and PUF was synthesized as a modified alginate-polyurethane composite (ALG-PUC) monolithic column situated in-valve of an FI set up with FlameAAS. With the setup (only very basic components of which were available for our work), applicability of the single standard calibration approach was possible. Although the sensitivity obtained from the system was not comparable with some other relatively expensive instruments, it demonstrated some benefits compared to the use of conventional FlameAAS alone, notably, that FlameAAS is commonly available in laboratories.

## Figures and Tables

**Table 1:** Correlation involving the use of single standard calibration.

Condition ^1^	Pb^2+^ (µg mL^−1^)	Loading Time (min)	Calculated µg Pb^2+^	FI Response (Absorbances)			
				I	II	III	Average
A	0.1	5	0.5	0.000	0.001	0.001	0.001 ± 0.000
B	0.2	5	1	0.006	0.004	0.004	0.004 ± 0.001
C	1.0	5	5	0.016	0.012	0.009	0.013 ± 0.003
D	2.0	5	10	0.020	0.022	0.015	0.019 ± 0.003
E	2.0	7	14	0.031	0.026	0.021	0.026 ± 0.004
F	2.0	10	20	0.035	0.030	0.041	0.035 ± 0.004
F	4.0	5	20	0.040	0.048	0.040	0.043 ± 0.004
G	4.0	7	28	0.057	0.053	0.058	0.056 ± 0.002
H	6.0	5	30	0.057	0.056	0.052	0.055 ± 0.002
I	8.0	5	40	0.069	0.072	0.068	0.070 ± 0.002
J	6.0	7	42	0.091	0.083	0.078	0.084 ± 0.005
K	10.0	5	50	0.099	0.093	0.092	0.095 ± 0.003
L	8.0	7	56	0.105	0.111	0.104	0.107 ± 0.003
M	4.0	15	60	0.120	0.111	0.123	0.118 ± 0.005
M	6.0	10	60	0.113	0.123	0.117	0.118 ± 0.004
N	10.0	7	70	0.138	0.121	0.127	0.129 ± 0.007
O	8.0	10	80	0.144	0.143	0.142	0.143 ± 0.001
P	6.0	15	90	0.175	0.172	0.169	0.172 ± 0.002
Q	8.0	15	120	0.224	0.201	0.220	0.215 ± 0.010
R	10.0	15	150	0.285	0.262	0.266	0.271 ± 0.010

^1^ Flow rate of loading at 1 mL min^−1^.

**Table 2:** Lead content in samples of aqueous sulfuric acid solutions contained in acid batteries.

Sample	Pb^2+^ (µg mL^−1^)			
	Added	Found	Recovery (%)	Content in Sample ^1^
S1	0.0	0.4 ± 0.1	-	2.0 ± 0.2
	2.0	2.5 ± 0.1	105	
S2	0.0	1.0 ± 0.1	-	5.0 ± 0.5
	2.0	3.0 ± 0.1	100	
S3	0.0	0.4 ± 0.1	-	2.0 ± 0.2
	2.0	2.6 ± 0.1	110	

^1^ The amount of Pb^2+^, µg mL^−1^ in three samples of aqueous sulfuric acid solutions contained in acid batteries = Found, Pb^2+^, µg mL^−1^ (calculated from calibration graph) × dilution factor (5 times).

## Data Availability

All the data are reported in this manuscript and Supplementary Materials.

## Supplementary Materials

The following are available online, Figure S1: IR spectra of sodium alginate (purple), PUF (blue) and ALG-PUC (green), Figure S2: SEM images with two different magnifications: (a1) PUF, 1000x; (a2) PUF, 5000x; (b1) ALG-PUC, 1000x; and (b2) ALG-PUC, 5000x (each sample was cut into a small piece, attached to carbon tape and received a sputtering coat with gold), Figure S3: Photos of: (a) PUF; (b) ALG-PUC; and (c) monolithic ALG-PUC rod, Figure S4: Full diagram of the work sequence, with each sample having a work loop of: a = loading time (varying as desire); b = washing time (1 min); c = eluting time (1 min); d = cleaning time (3 min); x1 and x2 = waiting time (0.5 min each), Table S1: Experimental results with loading time lower than 5 min.
