# Supplementary material for: Determination of Lead Employing Simple Flow Injection AAS with Monolithic Alginate-Polyurethane Composite Packed In-Valve Column
Source: Molecules. 2021 Jul 21;26(15):4397. doi: 10.3390/molecules26154397 (PMC8347769; doi:10.3390/molecules26154397)
Supplement: Supplementary file 1 [file molecules-26-04397-s001.zip › molecules-1288136-supplementary.pdf]

molecules

Article

Determination of Lead Employing Simple Flow Injection AAS with Monolithic Alginate-Polyurethane Composite Packed In-Valve Column

Piyanat Issarangkura Na Ayutthaya [1], Chonnipa Yeerum [1], Kullapon Kesonkan [1], Kanokwan Kiwfo [2], Kate Grudpan [2,*] Norio Teshima [3], Hiroya Murakami [3] and Monnapat Vongboot [1,*]

[1] Department of Chemistry, Faculty of Sciences, King Mongkut's University of Technology Thonburi, Bangkok, 10140, Thailand; piyanat.tp@gmail.com (P.I.); chonnipa.yeerum@gmail.com (C.Y.); kullapon.kesonkan@gmail.com (K.K.)
[2] Center of Excellence for Innovation in Analytical Science and Technology and Department of Chemistry, Faculty of Sciences, Chiang Mai University, Chiang Mai, 50200, Thailand; k.kanokwan11@gmail.com (K.K.)
[3] Department of Applied Chemistry, Aichi Institute of Technology, 1247 Yachigusa, Yakusa-cho, Toyota 470-0392, Japan; teshima@aitech.ac.jp (N.T.); hmurakami@aitech.ac.jp (H.M.)
* Correspondence: sumalee.tan@kmutt.ac.th (M.V.); Tel.: +662-470-8969, kgrudpan@gmail.com (K.G.); Tel.: +66-5394-1917

Supplementary Materials

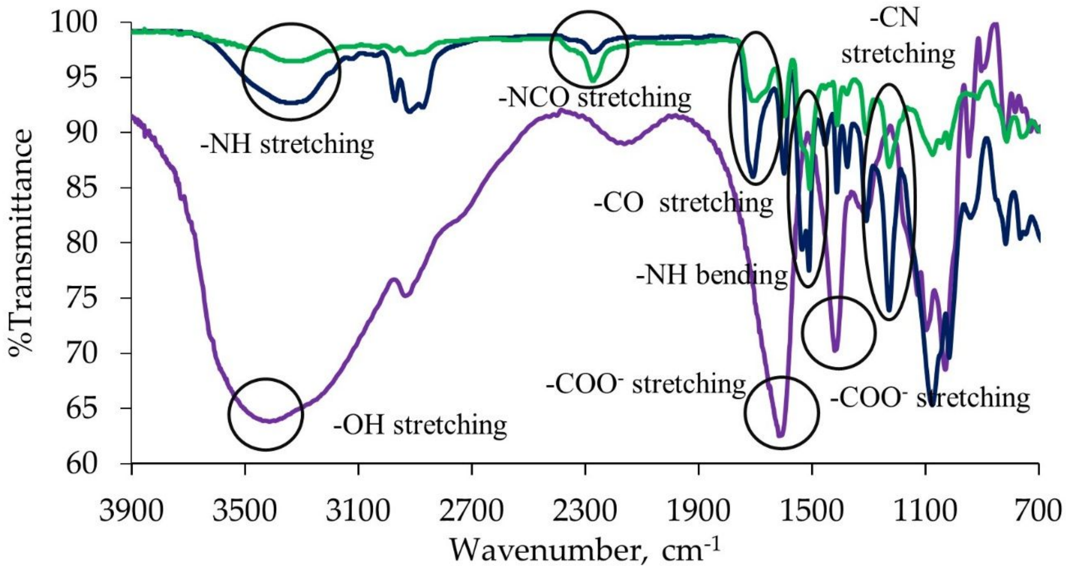

Figure S1. IR spectra of sodium alginate (purple), PUF (blue) and ALG-PUC (green).

The prepared PUF and ALG-PUC were ground into powder which was readily to be sensed by ATR-FTIR probe. Sodium alginate was treated similarly. From Figure S1, the -OH stretching at 3412 cm^{-1} of hydroxyl group for polymerization and -COO stretching of binding side to associate with Pb^{2+} ions at 1616, 1419 cm^{-1} were appeared from pure sodium alginate. From the IR spectra of unloaded PUF, -NH stretching and bending at 3341 and 1510 cm^{-1}, -CO and -CN stretching in 1708 and 1227 cm^{-1} indicated urethane bond of PU. However, NCO stretching still appeared owing to the ratio of 2:1 of MDI (-NCO) : Polyol (-OH). The groups of -NH stretching and bending at 3341 and 1510 cm^{-1}, and the stretching of -CO and -CN in 1708 and 1227 cm^{-1} implied the urethane bond of polyurethane. Apparently, -OH stretching of alginate disappeared and -CO stretching at 1708 cm^{-1} was broader than PUF due to overlapping of -COO^{-}

stretching from alginate. -NCO stretching at 2273 cm^{-1} still appeared owing to 2:1 of MDI (-NCO) : Polyol (-OH). Nonetheless, the difference between PUF and ALG-PUC was scarcely clear.

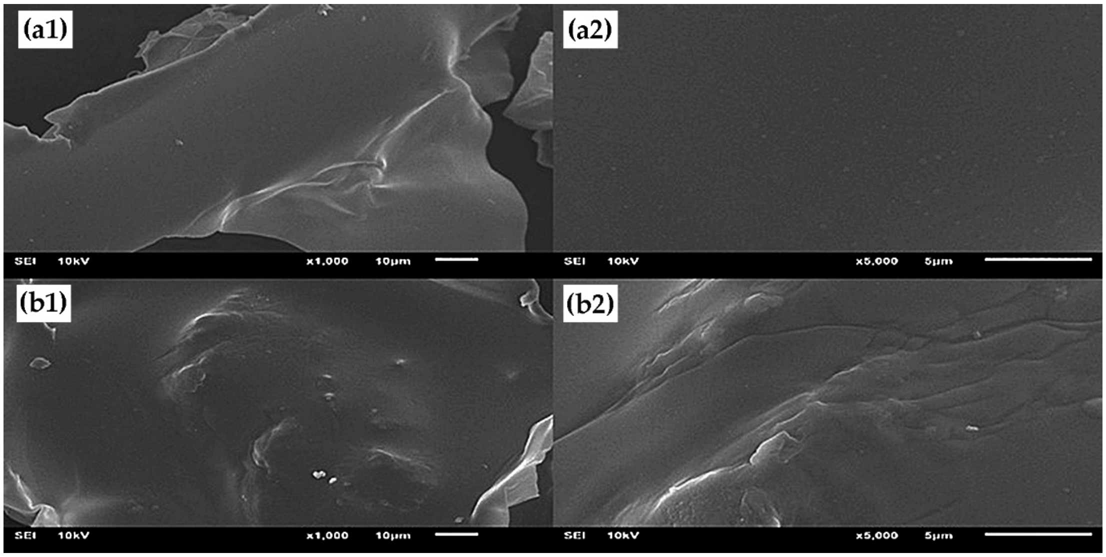

Figure S2. SEM images with two different magnifications: (**a1**) PUF, 1000x; (**a2**) PUF, 5000x; (**b1**) ALG-PUC, 1000x; (**b2**) ALG-PUC, 5000x (each sample was cut into a small piece, attached to a carbon tape and sputtering coat with gold).

Comparing the images of PUF and ALG-PUC at 1000x (a1 vs b1), roughness could be observed in b1. For more magnification, 5000x (the most powerful available for the experiment), roughness could be clearer seen in b2 while no roughness could be obsevred in a2. From the images, it is indicated that PUF exhibited flat surface while ALG-PUC.

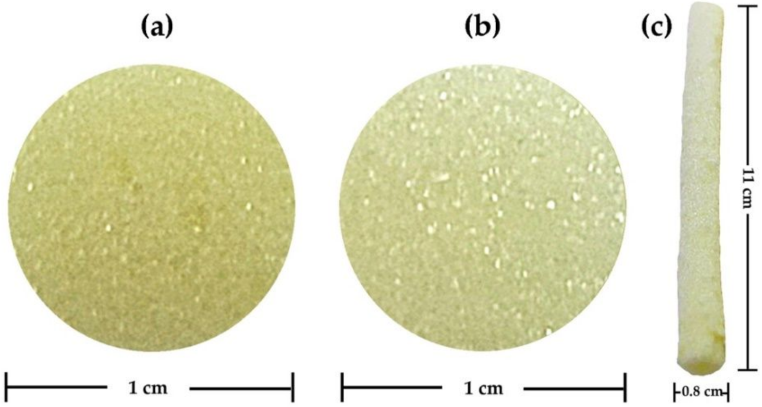

Figure S3. Photos of: (**a**) PUF; (**b**) ALG-PUC; (**c**) monolithic ALG-PUC rod.

Figure S3 depicts of PUF, ALG-PUC and monolithic ALG-PUC rod. From Figure S4, the ALG-PUC could be observed for its brightness and comparing to PUF and with better porosity.

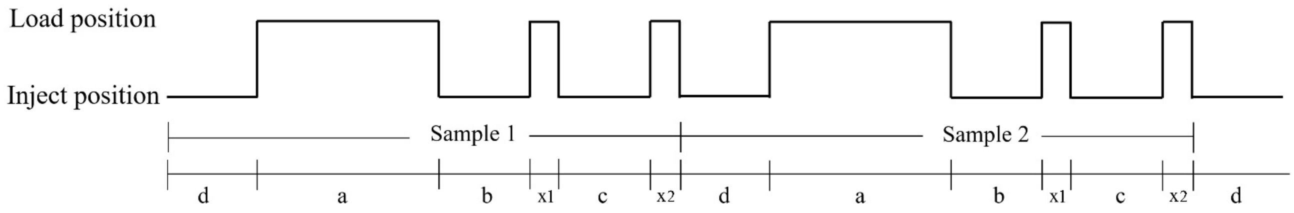

Figure S4. The full diagram of work sequence, each sample having a work loop of: a = loading time (varying as desire); b = washing time (1 min); c = eluting time (1 min); d = cleaning time (3 min); x1 and x2 = waiting time (0.5 min each).

Table S1. Experimental results with loading time lower than 5 min.

Condition [1]	Pb^{2+} (μg mL^{-1})	Loading time (min)	Calculated μg Pb^{2+}	FI response (absorbances)			
				I	II	III	average
1	2.0	2	4	0.002	0.003	0.004	0.003 ± 0.001
2	2.0	3	6	0.002	0.002	0.006	0.003 ± 0.002
3	4.0	2	8	0.006	0.007	0.008	0.007 ± 0.001
4	4.0	3	12	0.012	0.011	0.011	0.011 ± 0.000
5	6.0	2	12	0.009	0.009	0.009	0.009 ± 0.000
6	8.0	2	16	0.013	0.014	0.011	0.013 ± 0.001
7	6.0	3	18	0.029	0.029	0.021	0.026 ± 0.004
8	10.0	2	20	0.019	0.025	0.018	0.021 ± 0.003
9	8.0	3	24	0.048	0.039	0.037	0.041 ± 0.005
10	10.0	3	30	0.057	0.053	0.051	0.054 ± 0.002

[1] Flow rate of loading at 1 mL min^{-1}.
